# Puerarin Reversing Autophagy‐Lysosomal Dysfunction via Acid Sphingomyelinase Inhibition in Cardiomyocytes

**DOI:** 10.1111/jcmm.70427

**Published:** 2025-02-24

**Authors:** Yin‐ping Li, Qian He, Xiao‐ying Yu, Sheng‐tao Xiong, Hong‐jing You, Yue Xuan, Wei‐yan Liao, Ze‐yu Chen, Hai‐yan Li, Wei Wang, Yang Chen, Xiao Wang

**Affiliations:** ^1^ Research Centre of Basic Integrative Medicine, School of Basic Medical Sciences Guangzhou University of Chinese Medicine Guangzhou Guangdong P.R. China; ^2^ Department of Pharmacology, School of Pharmaceutical Guangzhou University of Chinese Medicine Guangzhou Guangdong China

**Keywords:** ASM, autophagy, heart failure, lysosome, puerarin

## Abstract

Heart failure (HF) is a major cardiovascular disorder characterised by high prevalence and mortality rate. Recent studies have emphasised the role of autophagy in development and progression of HF. Dysfunctions in lysosomes and autophagic processes are closely associated with the aetiology of HF. Puerarin (PUE), a traditional Chinese medicine known for its antioxidant, anti‐inflammatory and antiapoptotic properties, is widely used for the treatment of HF. However, the effectiveness of PUE in HF management via the modulation of autophagy requires further investigation. We used a mouse model of transverse aortic constriction to investigate protective effects of PUE on the autophagy–lysosomal pathway (ALP). We assessed heart function using echocardiography and performed histological staining for fibrosis and hypertrophy. RT‐qPCR for atrial natriuretic peptide (ANP)/brain natriuretic peptide (BNP) and Western blotting for p62/LC3/LAMP1/Beclin1 were performed. Immunofluorescence was used to identify the autophagosomes, autolysosomes and lysosomes. In addition, immunohistochemistry was performed to detect acid sphingomyelinase (ASM) and ceramides. ASM siRNA was transfected into cardiomyocytes to evaluate autophagy. PUE treatment significantly reduced myocardial fibrosis and hypertrophy in HF‐induced mice. PUE also effectively ameliorated ALP impairment in HF‐induced mice and H9c2 cells. Mechanistically, PUE restored lysosomal homeostasis by inhibiting ASM expression and lysosomal transport, thereby enhancing lysosomal activity. These results underscore the therapeutic potential of PUE in correcting the ASM‐mediated disruption of the HF‐linked autophagy–lysosomal pathway.

**Abbreviations**
ALP: autophagy‐lysosome pathway
ANP: atrial natriuretic peptide
ASM: acid sphingomyelinase
BNP: brain natriuretic peptide
BP: biological process
BW: body weight
CAP: captopril
CC: cell composition
CHF: chronic heart failure
CQ: chloroquine
EF: ejection fraction
FS: fractional shortening
HF: heart failure
HFD: high‐fat diet
HW: heart weight
LDH: lactic acid dehydrogenase
MF: molecular function
OGD: oxygen–glucose deprivation
PKCε: protein kinase C epsilon
PUE: puerarin
TAC: transverse aortic constriction
TFEB: transcription factor EB
UTP: uridine triphosphate
WGA: wheat germ agglutinin

## 1 Introduction

Heart failure (HF) is a major health concern, primarily arising from the progressive deterioration of cardiac function [1]. The central importance of myocardial remodelling in the development and progression of HF is widely acknowledged [2]. This remodelling involves structural and functional changes in the heart muscles that are triggered by various types of injuries. Myocardial hypertrophy and fibrosis are critical elements of this process [3].

Autophagy is a crucial lysosomal degradation pathway that plays a vital role in maintaining cellular function by discarding damaged organelles and proteins [4]. Autophagy is fundamental for sustaining myocardial integrity and ensuring normal heart function. It is a key adaptive response to cardiac injury and stress. Impairment of autophagic processes, especially obstruction of autophagic flux in cardiomyocytes, is strongly implicated in the onset of myocardial hypertrophy and fibrosis [5]. This underscores the potential of targeting autophagy as an effective strategy for HF prevention. The current research suggests existence of a complex relationship between acid sphingomyelinase (ASM) and autophagy regulation. When ASM is directed towards lysosomes, it can hinder the breakdown of autophagosomes, resulting in their accumulation and disruption of autophagic flux [6].

Puerarin (PUE), the primary active constituent of the Chinese herbal medicine *Pueraria lobata* , is obtained from the leguminous plants *Pueraria lobata* (Willd.) Ohwi and *Pueraria thomsonii* Benth [7]. These plants have a long history of application in traditional Chinese medicine [7]. Clinically, PUE is renowned for its vasodilatory properties and its ability to enhance circulation [8], making it particularly effective for treating cardiovascular and cerebrovascular ischemic conditions [9, 10]. Contemporary pharmacological studies have further revealed the potential of PUE in treating HF and enhancing cardiac function [11]; however, its underlying mechanism remains to be completely understood. Recent research has uncovered that PUE stimulates protective autophagy via the 14‐3‐3γ/PKCε pathway, thereby mitigating myocardial damage caused by lipopolysaccharide and doxorubicin [12, 13].

In the present study, we examined the contribution of PUE to myocardial protection. We focused on understanding its role in revitalising myocardial autophagy and increasing the autophagic flux. We also explored the involvement of the ASM pathway in this mechanism.

## 2 Materials and Methods

### 2.1 Chemicals and Antibodies

PUE (C_21_H_20_O_9_; molecular weight, 416.38; purity ≥ 98%; JOT‐10054) was obtained from Pufei De Biotech Co. Ltd. (Chengdu, China). The Captopril (CAP) and carboxymethylcellulose sodium salts were purchased from Sigma‐Aldrich (St. Louis, MO, USA). Chloroquine (CQ) was obtained from Meilun Bio (Dalian, China, MB1668‐1). Dulbecco's modified Eagle's medium (DMEM), DMEM (glucose free) and fetal bovine serum (FBS) were purchased from Gibco (CA, USA; C11995500BT, 11966025, 10270106). Antibodies against p62 were purchased from Proteintech (Wuhan, China; no. 66184‐1‐Ig). LC3 and Beclin1 were purchased from CST (Boston, USA; 3738S, 2775s), LAMP1 and β‐actin were purchased from Santa (Boston, USA; sc‐20011, sc‐47778). ASM was purchased from Abcam (Cambridge, UK; ab272729). Ceramides were obtained from ENZO (New York, NY, USA). Hoechst33342, DAPI and LysoTracker Red were purchased from Beyotime (Jiangsu, China). Wheat germ agglutinin conjugates (WGA) were purchased from AAT Bioquest (CA, USA). mRFP‐GFP‐LC3 adenovirus was obtained from Hanbio Biotechnology (Shanghai, China). ASM siRNA and its interference control (si‐NC) were obtained from Tsingke Biotechnology (Beijing, China). The ASM siRNA and primer sequences are listed in Appendix S1.

### 2.2 Animals and Experimental Protocols

All animal experiments were performed in accordance with the guidelines of the Laboratory Animal Ethics Committee of the College of Traditional Chinese Medicine, Guangzhou University of Chinese Medicine. All mice were maintained in a specific pathogen‐free facility under controlled temperature (20°C–26°C) and humidity (40%–70%) with a 12‐h light/dark cycle at the Laboratory Animal Room of the College of Traditional Chinese Medicine (Guangzhou, China; certificate no. SYXK 2019‐0202). Food and water were provided ad libitum. All animal studies were designed using randomised and blinded analyses to produce equally sized groups.

Male C57BL/6 wild‐type mice (7–9 weeks old; 20–30 g) were purchased from Guangzhou Ruige Biological Technology Co. Ltd. (Guangzhou, China; certificate no. SCXK 2021‐0059). For the HF model, TAC induction was established after 1 week of adaptive feeding. Mice were anaesthetised with 0.3% pentobarbital (0.1 mL/10 g; intraperitoneal injection), and their chest cavity was opened. Subsequently, a 6‐0 nylon suture was tied around the aorta, and a 26‐gauge needle was removed after ligation. The chest cavity was then sutured. The sham group underwent a similar procedure except for ligation. Two weeks after surgery, the surviving mice were separated randomly into six groups (*n* = 6 individuals/group): the sham group, TAC group, three treatment groups of TAC mice treated with PUE (20, 40 and 80 mg/kg; intragastric administration) and one group treated with CAP (30 mg/kg; intragastric administration). CAP is a positive drug. Mice in the sham and TAC groups were administered 0.5% carboxymethylcellulose sodium salt solution. All mice were continuously treated for 4 weeks (once daily).

### 2.3 Echocardiography

Echocardiography was used to record and assess cardiac function in mice at 1 and 6 weeks after the surgery. Mice were anaesthetised using isoflurane inhalation. An echocardiography Vevo 2100 imaging system was used to measure and analyse the ejection fraction (EF) and fractional shortening (FS) in the parasternal short axis using M‐mode and B‐mode imaging.

### 2.4 Organ Weight Analysis and Histological Analysis

The heart weight (HW)/body weight (BW) ratio was measured. After paraffin embedding, the heart tissue was sectioned into 4‐μm thick slices and stained with haematoxylin and eosin (HE) staining solution (Leagene, China) to assess morphological changes. Masson's trichrome stain (Leagene) was used to evaluate the degree of fibrosis under a microscope (Olympus, Tokyo, Japan). Furthermore, 6‐μm thick, OCT‐frozen sections were stained with WGA to assess the cross‐sectional area of cardiomyocytes in the heart tissue and the images were captured using a confocal microscope (Zeiss, Germany). All data were analysed using ImageJ software.

### 2.5 Immunohistochemistry

For immunohistochemistry analysis, as previously stated, paraffin sections of myocardial tissue (4‐μm thick) were dewaxed in water. Citrate buffer (pH 6.0) was used for antigen retrieval. Sections were blocked with 5% goat serum, and hydrogen peroxide was added to remove endogenous peroxidases. The sections were then incubated overnight at 4°C with primary antibodies for individual proteins: Ceramide and Anti‐ASM. The sections were then incubated at 20°C–25°C with anti‐mouse (Vector Laboratories, CA, USA) or anti‐rabbit (Vector Laboratories) secondary antibodies and HRP‐streptavidin (Boster, Wuhan, China). DAB chromogenic solution (Vector Laboratories) was used according to the manufacturer's instructions for colour development. All histopathological sections were visualised under a microscope, and quantification was performed using ImageJ software.

### 2.6 Biochemical Approaches to the Culture of H9c2 Cells and Establishment of an In Vitro Model of Oxygen–Glucose Deprivation

H9c2 embryonic rat cardiac cells, provided by Professor Yang Chen from Guangzhou University of Chinese Medicine, were cultivated in DMEM supplemented with 10% FBS at 37°C under a humidified atmosphere containing 5% CO_2_ and 95% air. To replicate myocardial ischemic injury in vitro, H9c2 cells were subjected to oxygen–glucose deprivation (OGD) as previously described. Once 80%–90% confluent, the culture medium was replaced with serum‐ or glucose‐free DMEM. Cells were incubated in a hypoxic chamber (95% N_2_, 5% CO_2_) at 37°C for 12 h. Cells in the control group were cultured in high‐glucose DMEM. PUE was dissolved in 0.1% DMSO and diluted to the concentrations of 5, 10 and 20 μmol/L. CQ and CAP were diluted to 10 and 1 μmol/L, respectively. All interventions were administered 12 h after the initiation of OGD.

### 2.7 Assessment of Cell Viability and Quantification of Lactate Dehydrogenase in Culture Media

Cell viability was assessed using the CCK8 assay (Biosharp, Hefei, China), and the activity of lactate dehydrogenase (LDH) released into the culture medium was determined using an LDH activity assay kit (Jiancheng Bioengineering Institute, Nanjing, China). Cell viability and LDH activity were measured according to the manufacturer's instructions.

### 2.8 Evaluation of Autophagic Flux by Monitoring LC3 Fluorescence Changes

Autophagic flux was evaluated by the mCherry‐EGFP‐LC3 assay, as previously described [14]. In brief, H9c2 cells were cultured in glass‐bottom cell culture dishes and transfected with EGFP‐mCherry‐LC3 adenovirus (Han Heng Biotechnology, Shanghai, China) at a MOI of 50 for 24 h. After transfection, cells were washed with PBS and observed under a laser scanning confocal microscope. EGFP is unstable in acidic lysosomes, whereas mCherry is stable. Co‐localization of red and green fluorescence indicated autophagosome induction. Red fluorescence without green overlay appeared as red puncta in the merged images, indicating autolysosome formation. Autophagic flux was evaluated by counting the number of dots per cell.

### 2.9 Western Blot Analysis

Protein samples were obtained from both mouse heart and H9c2 cells, and the total protein concentration was measured using a BCA assay. Equivalent amounts of protein were separated using SDS‐PAGE and blotted onto PVDF membranes. Membranes were blocked with 5% non‐fat milk for 1 h and incubated with primary antibodies at 4°C overnight. The following day, membranes were incubated with HRP‐conjugated secondary antibodies at 20°C–25°C. Protein bands were visualised via chemiluminescence, and quantitative analysis was performed using ImageJ software to assess the relative protein expression levels.

### 2.10 Immunofluorescence

H9c2 cells and mouse heart sections were fixed and permeabilized with Triton X‐100. After blocking with 5% milk, cells and tissues were incubated with p62‐, LC3‐, ASM‐ and ceramide‐targeting primary antibodies at 4°C overnight. Subsequently, incubated with fluorescence‐labelled secondary antibodies that specifically recognised primary antibodies, the cells and tissues were stained with DAPI to visualise the nuclei. Finally, they were examined under an inverted confocal microscope.

### 2.11 Real‐Time Quantitative Polymerase Chain Reaction (RT‐qPCR)

The total RNA of H9c2 cells was isolated using TRIzol (Thermo Fisher Scientific, Waltham, MA, USA), and its concentration was quantified using a Nanodrop 2000 (Thermo Fisher Scientific). Subsequently, complementary DNA synthesis was performed using a Hifair AdvanceFast 1st Strand cDNA Synthesis Kit (Yeasen Biotechnology, Shanghai, China) as per the manufacturer's instructions. The expression levels of atrial natriuretic peptide (ANP) and brain natriuretic peptide (BNP) mRNA were determined using the SYBR Green I assay [15]. Melting curve analysis was conducted to confirm the specificity of the amplified product. Primer sets were designed by Beijing Tsingke Biotech (Beijing, China). The relative expression levels are presented as $2^{-\Delta \Delta C_{t}}$ values.

### 2.12 Experiment for Staining Cells With LysoTracker Red

The H9c2 cells were seeded onto a 96‐well plate and allowed to grow to the desired confluence. The cells were then washed with PBS to remove any residual medium or debris. Next, the LysoTracker Red working solution was added to the cell culture mediumm, and the cells were incubated at 37°C for 30 min. After incubation, the nuclei were stained with Hoechst 33342 for visualisation. Finally, the stained cells were examined under a confocal microscope.

### 2.13 Transfection of H9c2 Cardiomyocytes With ASM siRNA

H9c2 cardiomyocytes were seeded onto 6‐well culture plates and cultured until 70% confluence was reached before transfection. In a sterile environment, siRNA and Lipo8000 were added to an appropriate amount of DMEM, followed by gentle mixing to allow for the full combination of siRNA and Lipo8000. The siRNA–Lipo8000 complex was then added to the cell culture medium to achieve even distribution on the cell surface. The cells were then placed in a 37°C, 5% CO_2_ incubator for cultivation. Twenty‐four hours after transfection, the cells were collected for subsequent experiments.

### 2.14 Collection of Active Components of PUE

The Traditional Chinese medicine platform of TCMSP database (https://old.tcmsp‐e.com/tcmsp.php) was used to search ‘Pueraria’ as the search term. Potential drug targets of PUE were screened from the search results and the UniProt database (https://www.uniprot.org/) was used for ID conversion.

### 2.15 Collection and Screening of Disease Targets

‘GeneCards database’ (https://www.genecards.org/) and ‘Chronic heart failure’ were used as search terms. The search results were then imported into Microsoft Excel for processing. Screening was performed on the condition that the ‘relevance score’ ≥ median. Moreover, ‘OMIM database’ (https://omim.org/) was used to conduct a search in the ‘gene map’ section with ‘Chronic heart failure’ as the search term, and the search results were brought to Microsoft Excel for data processing. Target information obtained from the two databases was combined to remove duplicate targets.

### 2.16 Construction of Potential Target Protein Interaction Network of PUE–Chronic Heart Failure

The potential targets of PUE and target information of chronic heart failure (CHF) were imported into ‘FUNRICH3.1.3’ software to make Venn diagram, and the common targets of PUE and CHF were obtained, which were introduced into the ‘STRING’ platform (https://cn.string‐db.org/) for protein–protein interaction analysis.

### 2.17 GO Functional Enrichment Analysis and KEGG Pathway Enrichment Analysis

The intersection targets of PUE and CHF were imported into the ‘Metascape’ database (https://metascape.org/gp/#/main/step1) for KEGG pathway and GO function enrichment analyses. The ‘Wei Sheng Xin’ website (https://www.bioinformatics.com.cn) was used for data visualisation.

### 2.18 Molecular Docking

The structures of receptor proteins and ligand small‐molecule compounds were obtained from the PDB and PubChem databases, and then, the ligand molecules were converted into pdbqt format using OpenBabel (version 3.1.1). The receptors were pre‐treated with AutoDock Tools1.5.6, such as dewatering and hydrogenation. The range parameters for molecular docking were set using a grid plate, the docking mode was set as ‘semi‐flexible docking’, and the docking algorithm was set as the Lamarck genetic algorithm. Molecular docking was performed using AutoDock Vina (version 1.1.2), and the molecular docking results were visualised using PyMOL software (version 2.20).

### 2.19 Statistical Analysis

Data are presented as mean values ± standard deviation (SD). One‐way analysis of variance (ANOVA) was performed using SPSS (version 27.0) to determine significant differences among multiple groups. Statistical significance was set at *p* < 0.05.

## 3 Result

### 3.1 Protective Mechanism of PUE Against HF Triggered by Pressure Overload

**FIGURE 1:**
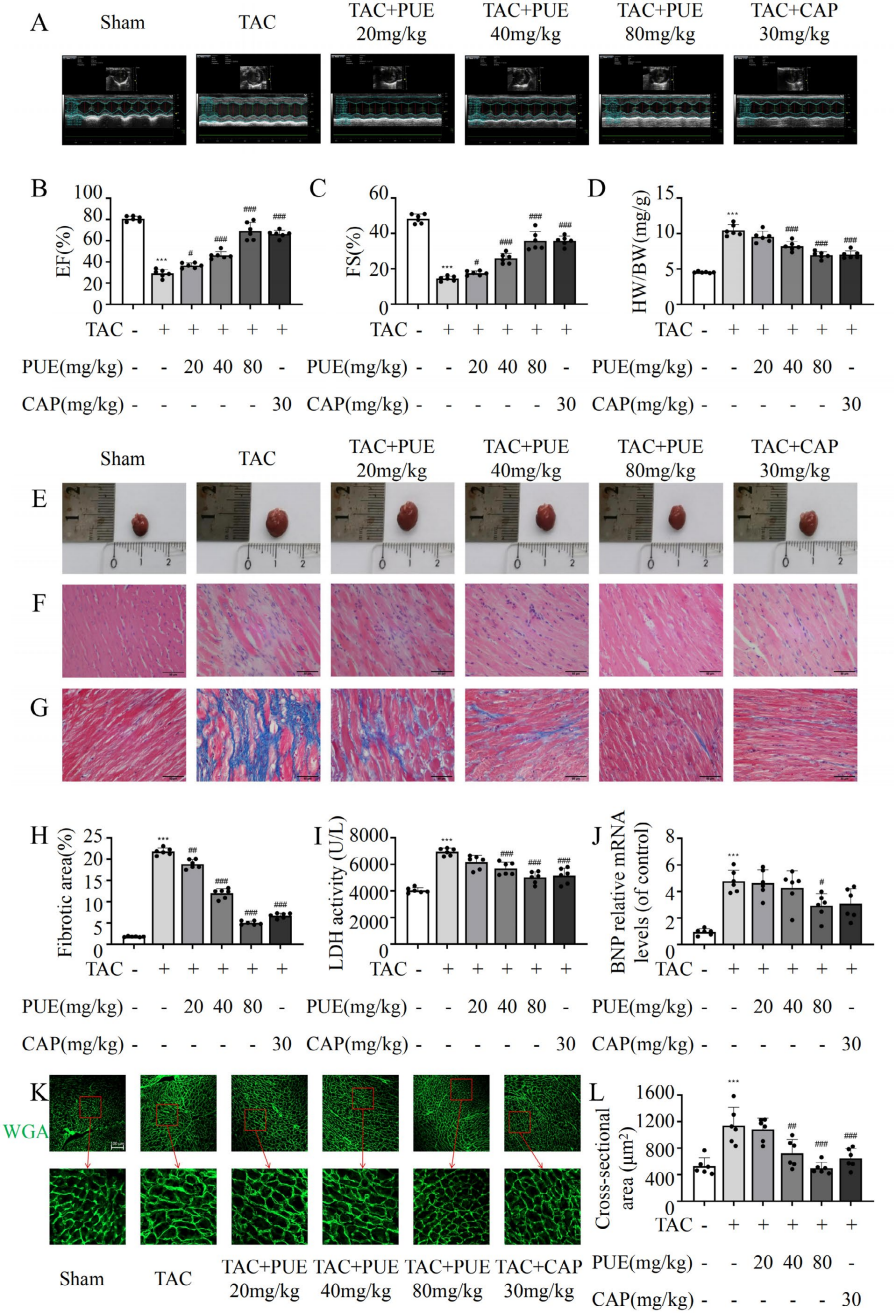
The effects of PUE on cardiac function in TAC mice. (A) Representative images of M‐mode echocardiography at the sixth week after TAC surgery. (B, C) Echocardiographic parameter analysis include EF and FS. (D) The ratios of heart weight to body weight (HW/BW). (E) General photos of heart in mice. (F) Representative images of HE‐stained in detail (scale bar: 50 μm). (G) The images of Masson's trichrome staining Quantification of fibrotic area (scale bar: 50 μm). (H) Quantification of fibrotic area in G. (I) The LDH levels in mice of serum after TAC with different concentrations of PUE and CAP. (J) The mRNA level of BNP in cardiomyocytes after TAC with different concentrations of PUE and CAP. (K, L) WGA staining and the quantitative analysis of cardiomyocyte cross‐sectional area. *p < 0.05, **p < 0.01, ***p < 0.001 versus the sham group (*n* = 6); ^#^ *p* < 0.05, ^##^ *p* < 0.01, ^###^ *p* < 0.001 versus the TAC group (*n* = 6). Data are presented as the mean ± SD.

Six weeks after surgery, M‐mode echocardiography was performed to detect cardiac function in all experimental mice. Figure 1A shows the representative echocardiography in the six groups of mice, which indicated left ventricular dilation in all mice except in the sham group. As shown in Figure 1B,C, the EF and FS values of the model group were significantly lower than those of the sham group, indicating that the ventricular wall was thickened, the heart cavity was expanded, and heart function was impaired. EF and FS were improved by low and medium doses of PUE to different degrees and were significantly improved in the high‐dose and CAP groups. The echocardiogram results showed that different doses of PUE ameliorated heart function in TAC mice, with the high‐dose group showing the most significant effect.

Myocardial remodelling, including myocardial hypertrophy and fibrosis, is an important pathological change in HF. To evaluate the effect of PUE on cardiac hypertrophy, the hearts of the mice were weighed and measured. Six weeks after surgery, the hearts of mice in the TAC group were significantly enlarged compared to those in the sham group and recovered after PUE or CAP intervention (Figure 1E). The HW/BW ratio was significantly high in the TAC group, which was significantly alleviated by daily PUE or CAP treatment (Figure 1D). Masson's trichrome and HE staining were used to detect the effect of PUE on morphological damage, and these results showed severe pathological changes in the heart tissues of the TAC group, including myocardial arrangement disorder, muscle fibre rupture and collagen fibre hyperplasia. PUE and CAP clearly changed in the cardiac tissue (Figure 1F–H). WGA staining and quantitative analyses further validated the role of PUE in myocardial hypertrophy. The cross‐sectional area of the cardiomyocytes in the TAC group was larger than that in the sham group, which was alleviated by PUE or CAP treatment (Figure 1K,L). To assess myocardial injury in mice, we measured the serum LDH levels. Compared to the sham group, the LDH level in the TAC group was significantly higher and was significantly lower in the middle‐ and high‐dose PUE and CAP groups compared to the TAC group (Figure 1I). In addition, RT‐qPCR showed that the mRNA expression level of BNP significantly increased in the TAC group but decreased in a dose‐dependent manner after treatment with PUE (Figure 1J).

### 3.2 PUE Counteracts Autophagy Suppression Resulting From TAC

**FIGURE 2:**
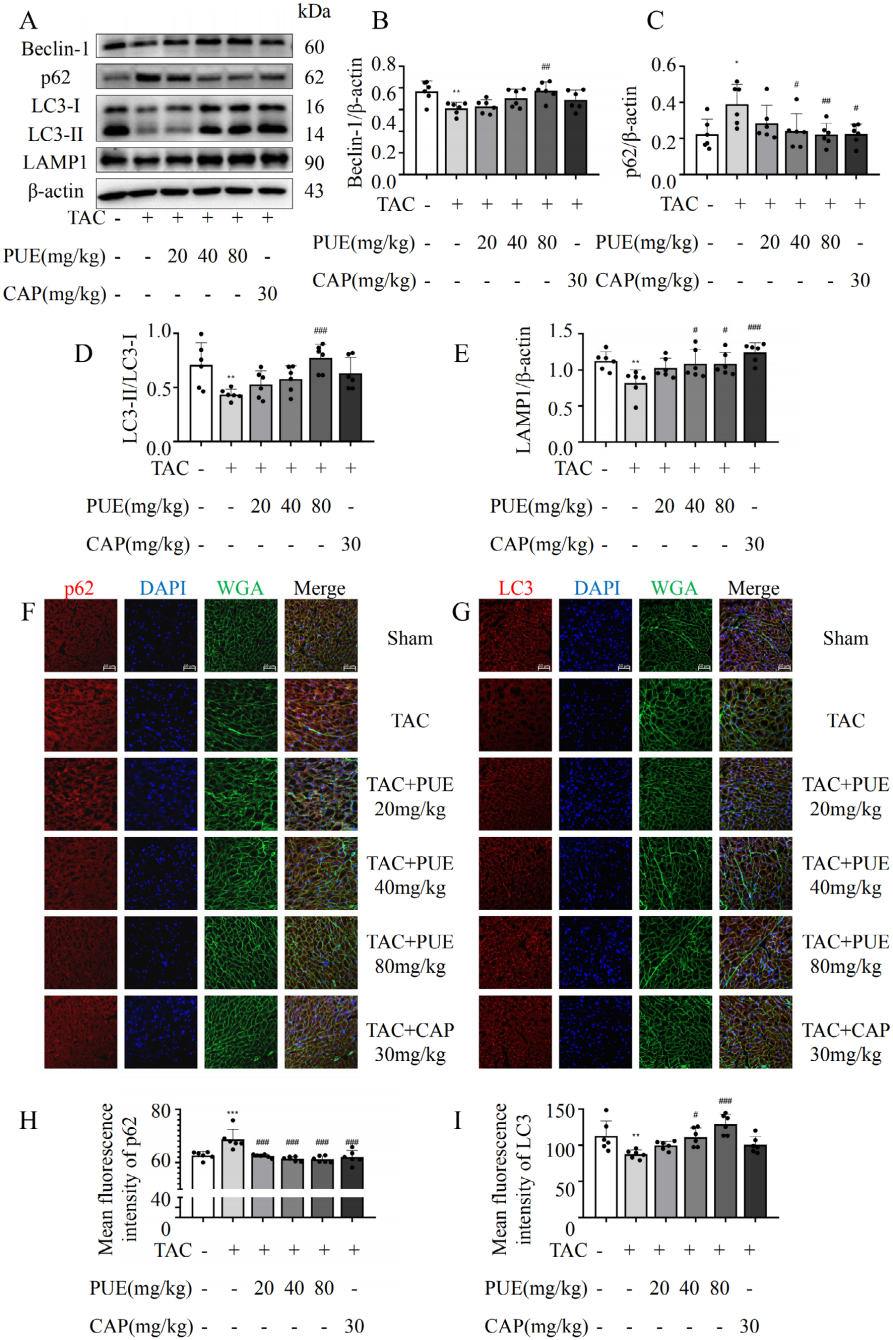
Effects of PUE on autophagy in TAC mice. (A) Western blot detection of Beclin1, p62, LC3 and LAMP1 protein expression. (B–E) Statistical graphs of protein expression after correction of grayscale values in A. (F, G) Immunofluorescent labelling of proteins p62 and LC3, DAPI labelling of cell nuclei, WGA labelling of myocardial cell membranes. (H, I) Average fluorescence intensities of p62 and LC3. *p < 0.05, **p < 0.01, ***p < 0.001 versus the sham group (*n* = 6); ^#^ *p* < 0.05, ^##^ *p* < 0.01, ^###^ *p* < 0.001 versus the TAC group (*n* = 6). Data are presented as the mean ± SD.

Abnormal autophagy activity may affect cardiac hypertrophy [16]. Therefore, we examined the autophagy levels in the TAC model. We observed a significant decrease in the protein expression of autophagy markers (LC3‐II and Beclin‐1) and an increase in p62 expression in TAC hearts. After treatment with PUE, autophagy inhibition occurred to some extent, and autophagy flux increased (Figure 2A–D). Because of the presence of non‐cardiomyocytes in myocardial tissue, we employed immunofluorescence to observe autophagy in the heart [17]. The immunofluorescence staining also showed the same trend (Figure 2F–I). To further investigate the mechanism underlying abnormal autophagic activity, we examined the function of lysosomes, which are closely associated with autophagy. We found that the expression of the lysosome function protein LAMP1 decreased in the TAC group but returned to normal levels after treatment with PUE in a concentration‐dependent manner (Figure 2A,E). This inspired us to investigate whether PUE regulates cardiac hypertrophy through the autophagy–lysosomal pathway (ALP) function, thereby restoring cardiac function and protecting against HF. In addition to autophagy, we investigated the roles of pyroptosis and apoptosis in programmed cell death. Our findings revealed that PUE did exert a significantly ameliorated TAC‐induced cellular juxtaposition and apoptosis in HF mice. This observation was based on the results of the TUNEL assay and immunofluorescence analysis of the juxtaposition proteins GASMD and CASPASE1 (Figure S1A–D), which is consistent with previous reports [18, 19, 20].

### 3.3 Protective Influence of PUE on Myocardial Cells Against OGD‐Led Injury

**FIGURE 3:**
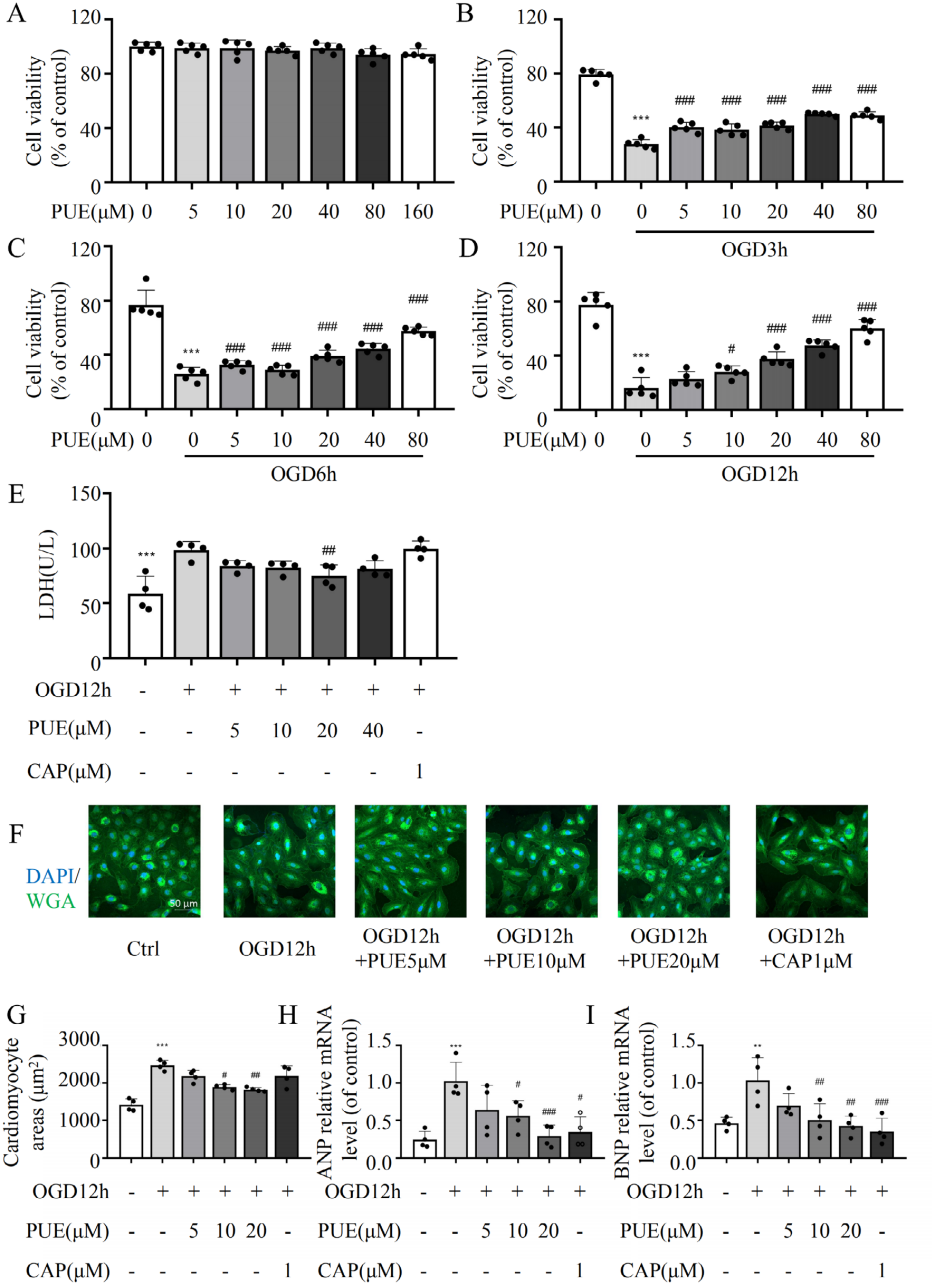
Effects of PUE on cardiomyocyte injury induced by OGD in vitro. (A) CCK‐8 assay was used to detect the effect of different concentrations of PUE on cardiomyocyte viability. (B–D) The effect of different concentrations of PUE on cardiomyocyte viability at different OGD time points. (E) After 12 h of OGD, cardiomyocytes were incubated with different concentrations of PUE and CAP, and LDH release was measured. (F) After 12 h of OGD, cardiomyocytes were incubated with different concentrations of PUE and CAP, and cell area was measured. (G) Statistical graph of cardiomyocyte area in F. (H, I) RT‐qPCR was used to detect the mRNA levels of ANP and BNP in cardiomyocytes after 12 h of OGD with different concentrations of PUE and CAP. *p < 0.05, **p < 0.01, ***p < 0.001 versus the control group (*n* = 4–5); ^#^ *p* < 0.05, ^##^ *p* < 0.01, ^###^ *p* < 0.001 versus the OGD12h without any drugs administered group (*n* = 4–5). Data are presented as the mean ± SD.

To assess the protective effect of PUE against HF by restoring autophagy suppression, we conducted an in vitro experiment using H9c2 cells to verify the effect of PUE on autophagy reduction (Figure 3A). Upon treating cells with different concentrations of PUE under OGD for different durations, cell viability recovered in a concentration‐dependent manner (Figure 3B–D). Based on previous research [21], we determined the concentration of PUE used to treat the cells and the duration of the OGD treatment. We observed that in the presence of OGD, H9c2 cells underwent cell damage with increased LDH release and atrial ANP and BNP mRNA levels, which serve as markers of HF (Figure 3E,H,I). Cell morphology also changed with an increase in the cell cross‐sectional area, as observed in the animal models (Figure 3F,G). However, upon treatment with PUE, LDH release decreased, ANP/BNP mRNA levels decreased and the cells recovered their normal morphology. These results suggest that PUE exerts a protective effect on myocardial cells and prevents myocardial hypertrophy in vitro.

### 3.4 PUE Reverses Autophagy Flux Impairment Caused by OGD Through Lysosomal Pathway

**FIGURE 4:**
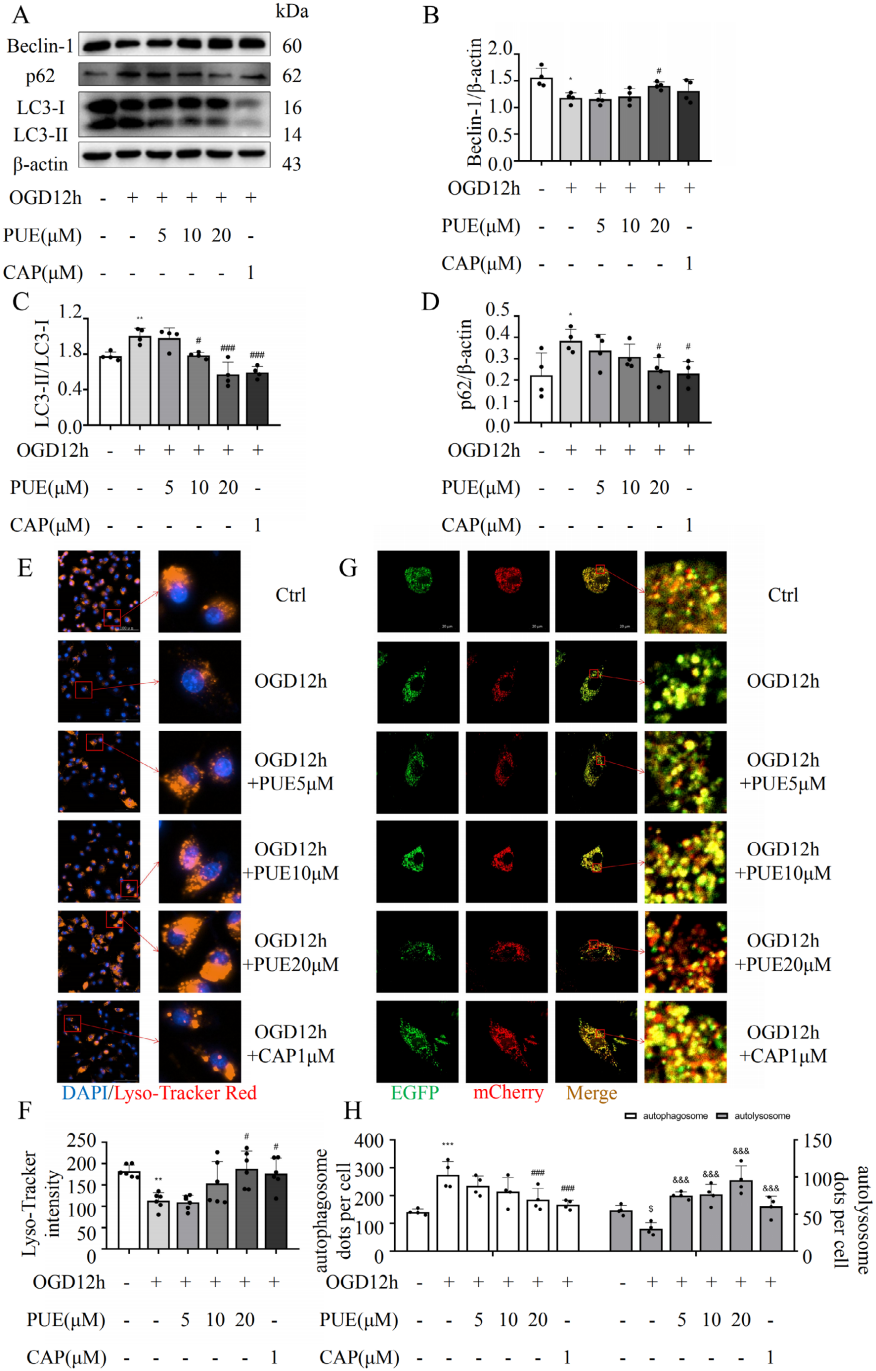
Effects of PUE on autophagy in cardiomyocytes subjected to oxygen–glucose deprivation in vitro. (A) Western blot analysis of Beclin1, p62 and LC3 protein expression. (B–D) Quantification of protein expression levels in A after correction of grayscale values. (E, F) Detection of lysosomal damage using Lyso‐Tracker Red staining, and quantification of the average fluorescence intensity. (G, H) Detection of autophagic flux using EFP‐mCherry‐LC3 adenovirus to transfect H9c2 cardiomyocytes, and quantification of autophagosomes and autolysosomes. In H, *p < 0.05, **p < 0.01, ***p < 0.001 versus the control group (*n* = 4) in autophagosomes; ^#^ *p* < 0.05, ^##^ *p* < 0.01, ^###^ *p* < 0.001 versus the OGD12h with any drug administered group (*n* = 4) in autophagosomes; ^$^ *p* < 0.05, *p* < 0.01, ^$^ *p* < 0.001 versus the control group (*n* = 4) in autolysosomes; ^&^ *p* < 0.05, ^&&^ *p* < 0.01, ^&&&^ *p* < 0.001 versus the OGD12h without any drug administered group (*n* = 4) in autolysosomes. In other figure, *p < 0.05, **p < 0.01, ***p < 0.001 versus the control group (*n* = 4); ^#^ *p* < 0.05, ^##^ *p* < 0.01, ^###^ *p* < 0.001 versus the OGD12h with any drug administered group (*n* = 4). Data are presented as the mean ± SD.

The effects of PUE on autophagy in H9c2 cells undergoing OGD were further investigated in vitro; the results were similar to those observed in the animal experiments. We initially employed CQ concentrations ranging 25–75 μM, in conjunction with various treatment durations, to assess the impact of CQ on autophagic flux in H9c2 cells. A direct correlation was noted between the CQ concentration and the magnitude of autophagic flux inhibition. Notably, the accumulation of LC3 preceded any significant alteration in the p62 levels after 6 h of CQ exposure. However, after 12 and 24 h of treatment, both p62 and LC3‐II accumulation were markedly elevated (Figure S2A–C). Based on these findings, we opted for a 12‐h treatment period with 25 μM CQ as the experimental condition for subsequent studies. Compared with the OGD group, autophagy levels were restored in PUE‐treated cells (Figure 4A–D). Remarkably, without the addition of the lysosome degradation inhibitor CQ, the autophagosome markers LC3 and p62 accumulated in the OGD group, but gradually decreased following PUE treatment. However, CQ treatment reversed this trend (Figure S3A–C), indicating that abnormal autophagic activity in myocardial cells undergoing ischemia and hypoxia may be related to lysosomal dysfunction (Figure S3D–F). We used LysoTracker Red to evaluate lysosome levels and found that OGD reduced lysosomal function as compared to that observed in the normal group (Figure 4E,F), supporting our hypothesis. Additionally, we observed that PUE treatment restored autophagic flux and accelerated autophagosome trafficking, which we believe is closely related to the PUE‐led restoration of lysosomal function (Figure 4G,H).

### 3.5 PUE Modulates the ASM/Ceramide Signalling Pathway in HF

**FIGURE 5:**
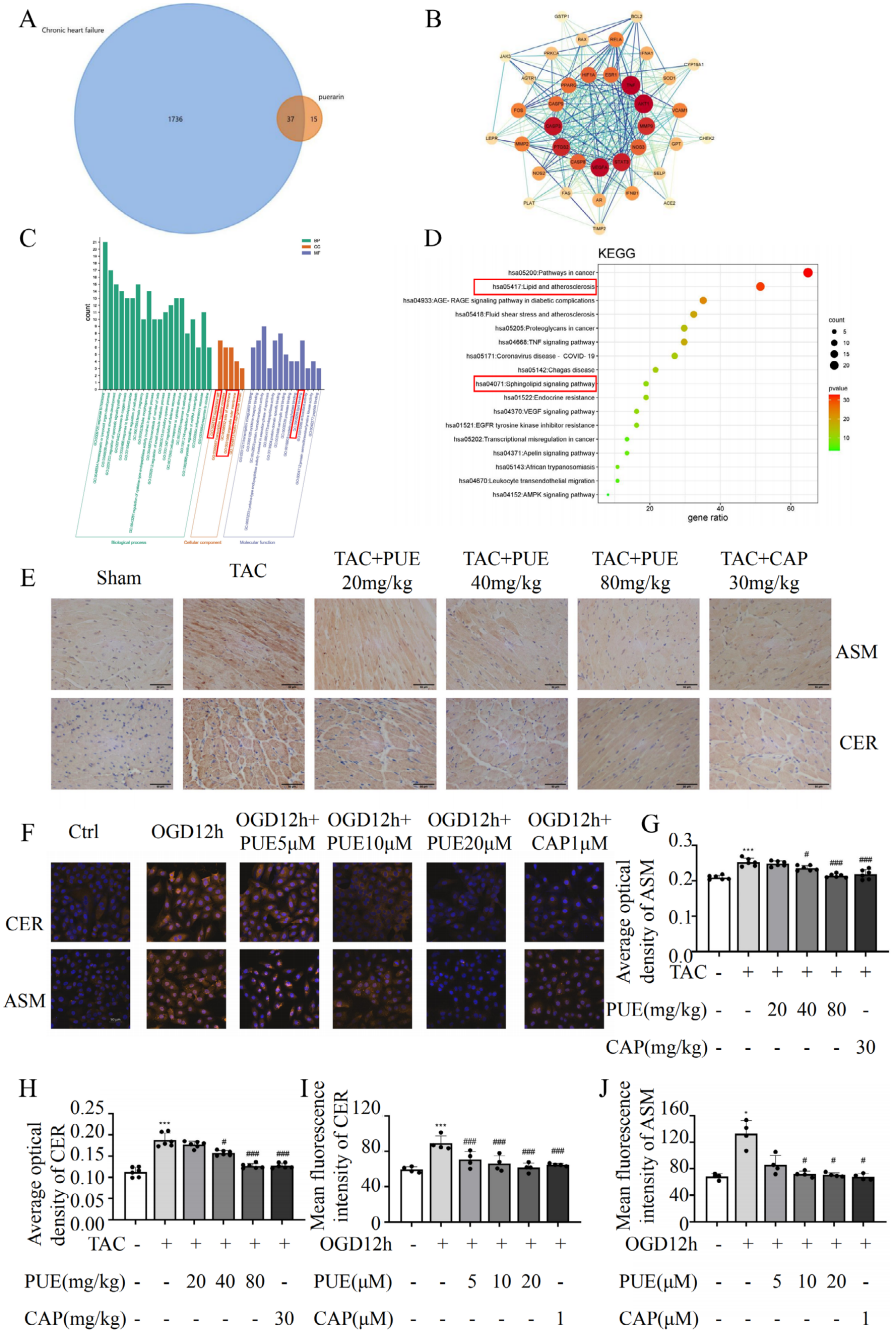
Regulation of sphingolipid metabolism in heart failure by PUE. (A) Venn diagram of the targets of PUE and chronic heart failure. (B) Analysis of protein–protein interaction on the common target of PUE and chronic heart failure. (C) Enrichment information of signalling pathways involved in the targets of PUE and chronic heart failure. (D) GO functional enrichment analysis of PUE‐chronic heart failure targets. (E) IHC analysis of the expression of CER and ASM in heart tissues and the quantitative analysis of average optical density in positive area. (F) Immunofluorescence detection of the expression of CER and ASM in cardiomyocytes treated with different concentrations of PUE and CAP in vitro. (G, H) Quantitative analysis of the expression of CER and ASM in heart tissues. (I, J) Quantitative analysis of the expression of CER and ASM in cardiomyocytes treated with different concentrations of PUE and CAP in vitro. *p < 0.05, **p < 0.01, ***p < 0.001 versus the sham group (*n* = 6); ^#^ *p* < 0.05, ^##^ *p* < 0.01, ^###^ *p* < 0.001 versus the TAC group (*n* = 6) in G and H. *p < 0.05, **p < 0.01, ***p < 0.001 versus the control group (*n* = 4); ^#^ *p* < 0.05, ^##^ *p* < 0.01, ^###^ *p* < 0.001 versus the OGD12h without any drugs administered group (*n* = 4) in I and J. Data are presented as the mean ± SD.

To elucidate the molecular mechanism by which PUE acts on HF, we conducted a network pharmacological analysis. The 52 potential action targets of PUE collected from the ‘TCMSP’ database and 1773 targets of CHF after selection were imported into the Funrich software (version 3.1.3) to generate a Venn diagram, and 37 common genes of PUE and CHF were obtained (Figure 5A). The common target proteins of PUE and CHF were introduced into the ‘STRING’ platform, and a protein–protein interaction network was initially constructed with 37 nodes and 326 connections. Thereafter, we imported the file into ‘Cytoscape’ (version 3.8.2) to adjust the image. The nodes were adjusted according to the degree value, with darker colours and larger nodes indicating proteins that interact more extensively with other proteins. The thickness and colour depth of the lines were adjusted according to the ‘Combined _score.’ Thicker lines and darker colours indicate a more extensive interaction network between proteins (Figure 5B). The 37 intersection targets of PUE–CHF were imported into ‘Metascape’ database for KEGG pathway and GO function enrichment analyses. GO functional enrichment analysis was performed in three parts: molecular function (MF), biological process (BP) and cell composition (CC). In terms of GO MF, lipid binding ranked in the top 10, and the first, third and fourth positions were all related to cell membranes in GO CC (Figure 5C). KEGG pathway enrichment analysis showed that PUE acted on CHF mainly through 20 pathways, of which the sphingolipid signalling pathways were ranked ninth (Figure 5D). To verify the results of the network pharmacology, we tested them experimentally using heart sections from mice and H9c2 cells using immunohistochemistry and immunofluorescence. The results showed that the levels of ASM and ceramide in the TAC group were significantly higher than those in the sham group, which were relieved after PUE and CAP treatment in H9c2 cells and mice (Figure 5E–J).

### 3.6 PUE Shields the Heart From HF Through ASM‐Mediated Regulation of the Autophagy–Lysosomal Pathway

**FIGURE 6:**
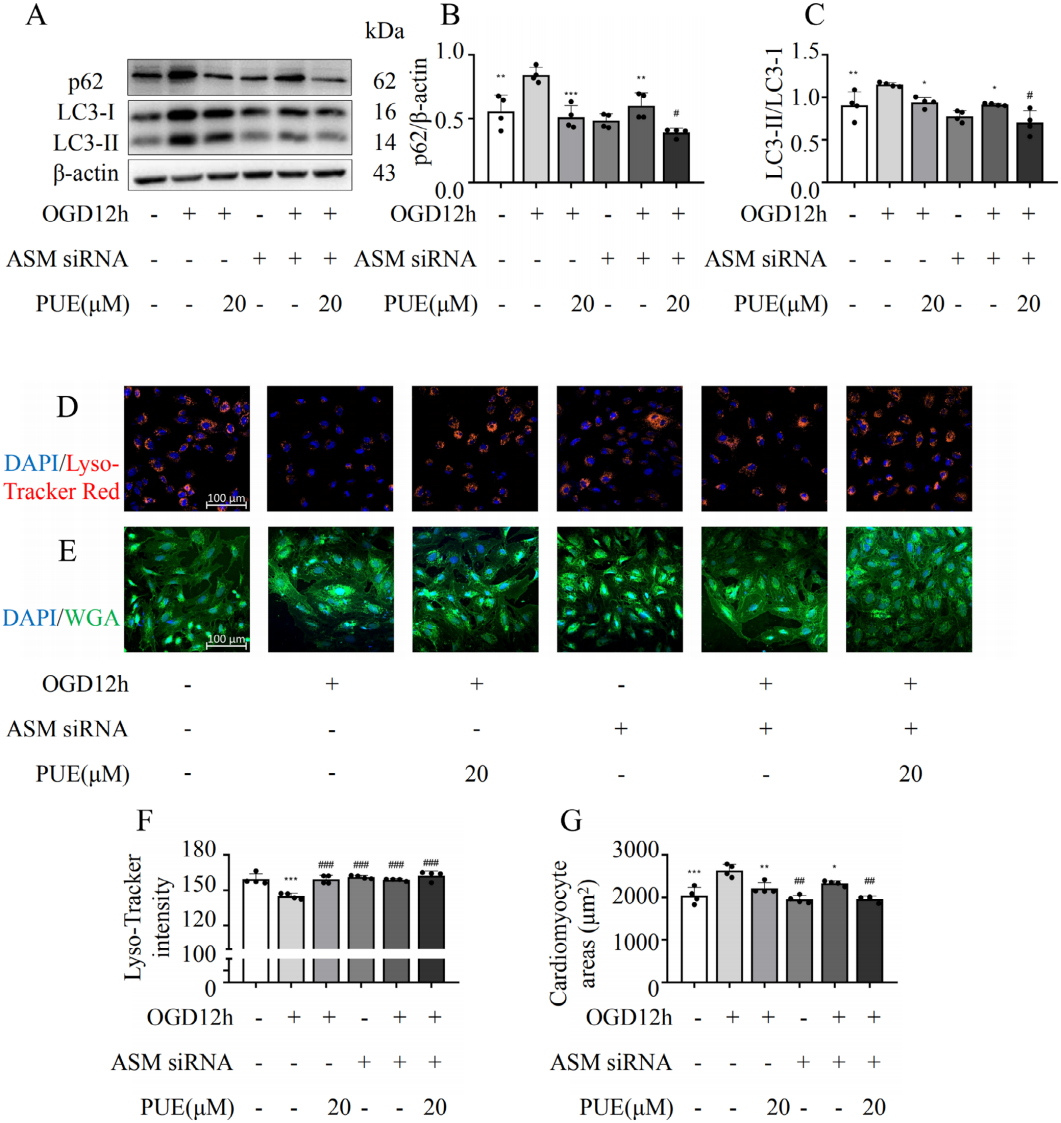
Knockdown of ASM siRNA accelerates autophagy flux. (A) Expression of p62 and LC3 after ASM siRNA knockdown. (B, C) Statistical graphs of A. (D–G) Fluorescent staining of myocardial cell area and Lyso‐Tracker Red after ASM siRNA knockdown and its statistical graph. *p < 0.05, **p < 0.01, ***p < 0.001 versus the OGD12h without any drugs administered group (*n* = 4); ^#^ *p* < 0.05, ^##^ *p* < 0.01, ^###^ *p* < 0.001 versus the OGD12h with ASM siRNA and no drugs administered group (*n* = 4). Data are presented as the mean ± SD.

Based on previous studies, we found that PUE restores autophagy in HF and regulates ALP function. Additionally, we demonstrated the role of PUE in the ASM/Ceramide signalling pathway in HF. To further explore the relationship between PUE and ASM, we investigated the co‐localization of ASM with lysosomes, as LAMP1 serves as a functional protein marker for lysosomes. Our findings from Figure S4A suggest that OGD‐treated cardiomyocytes exhibited increased co‐localization of ASM with LAMP1, whereas this co‐localization was attenuated following PUE treatment. In addition, we examined the nuclear translocation of transcription factor EB (TFEB), which regulates lysosomal function. Notably, we observed that the nuclear translocation of TFEB was reduced in response to OGD, leading to the inhibition of autophagy‐ and lysosome‐related gene transcriptional expression. However, PUE treatment restored nuclear translocation of TFEB (Figure S4B). These findings provide insight into the regulatory mechanisms underlying the effects of PUE on lysosomal function and autophagy in cardiomyocytes. Next, we utilised ASM siRNA in H9C2 cardiomyocytes. Transfection of ASM siRNA and treatment with PUE (20 μM) under an OGD environment revealed that reducing ASM expression accelerates autophagosome trafficking, reduces autophagosome accumulation and enhances autophagic flux, consistent with previous reports of ASM regulation of autophagy (Figure 6A–D,F). Furthermore, we observed that the downregulation of ASM expression increased TFEB nuclear translocation (Figure S4B) and prevented myocardial hypertrophy, an effect that was enhanced when combined with PUE treatment (Figure 6E,G). Molecular docking analyses revealed that PUE exhibited a strong binding affinity towards ASM (Figure S5A). Collectively, these results suggest that PUE protects the heart from HF by regulating the ALP via ASM.

## 4 Discussion

HF, a clinical syndrome associated with poor prognosis, has recently gained attention as a potential therapeutic target for reversing or delaying its myocardial remodelling [22]. Myocardial remodelling refers to changes in both cardiac myocyte loss and hypertrophy of myocardial cells, as well as the corresponding changes in non‐myocardial cells and the extracellular matrix [23, 24]. Pathological cardiac hypertrophic remodelling is often accompanied by cardiomyocyte autophagy, apoptosis or necrosis, among which deficiency or excessive cardiomyocyte autophagy is believed to be associated with adverse cardiac remodelling [5]. Autophagy is considered a protective mechanism that maintains the structure and function of the heart. Low levels of autophagy maintain myocardial cell homeostasis and are upregulated under stress overload, limiting the accumulation of misfolded proteins, mitochondrial dysfunction and oxidative stress [5]. Deletion of the autophagy gene *ATG14* reportedly results in a shortened lifespan and cardiac hypertrophy [25]. Additionally, a separate investigation focusing on the role of 21 ATG genes in cardiovascular diseases established a correlation between single nucleotide polymorphisms and pathological manifestations and functional deficits in cardiovascular diseases [26]. These findings suggest that autophagy could serve as a potential target for intervention in myocardial remodelling. Drugs that promote autophagy in myocardial cells, such as rapamycin, sirtuin activators, UTP, dinitroazole and ranolazine, can exert significant anti‐hypertrophic effects in myocardial hypertrophy models [27]. In this study, we used PUE, a plant‐derived compound, to treat a mouse model of HF. The results showed that PUE treatment effectively improved the heart function in mice and significantly restored multiple echocardiographic indices (Figure 1A). In addition, the treatment reduced myocardial fibrosis and cardiomyocyte hypertrophy and delayed myocardial remodelling (Figure 1B,C). These effects were also verified in in vitro cell experiments (Figure 3A–G).

Growing evidence suggests that PUE is a potentially effective drug for the treatment of HF via the autophagic pathway. PUE treatment can effectively restore autophagy inhibition in rats after aortic banding surgery and in isoprenaline‐induced H9c2 cells, thereby reducing myocardial cell hypertrophy and apoptosis [28]. Recent studies have found that PUE can induce adaptive autophagy and protect the heart from LPS‐induced injury by activating the 14‐3‐3γ/PKCε pathway [12], whereas another study reported that PUE protects cardiomyocytes from ischemia–reperfusion injury by inhibiting autophagy through the Akt signalling pathway [29], inhibiting collagen secretion and reducing myocardial fibrosis [30]. The bidirectional regulation of autophagy by PUE may be related to the timing of intervention. In this study, we demonstrated that PUE intervention restored the level of autophagy in a TAC‐induced mouse model of HF, enhancing autophagy flux, which is closely associated with lysosomal involvement in the autophagy process (Figure 2A–I). Lysosomes are the main sites for the degradation and recycling of macromolecules delivered by autophagosomes, and the intact function of lysosomes is an indispensable link in the autophagy degradation process [31]. We found that autophagy defects in HF were associated with decreased lysosomal expression, and PUE intervention reduced lysosomal damage, promoted the binding of autophagosomes and lysosomes and ensured smooth autophagy (Figure 4A–H).

Based on network pharmacology and experimental results, we found that PUE could regulate sphingolipid metabolism, especially ASM, in HF (Figure 5E,F). ASM is a sphingolipid hydrolase that functions in acidic environments and is a key lysosomal enzyme, closely related to lysosomal function [32]. As early as 2014, researchers proposed that ASM is a new regulator of autophagy, and partial genetic inhibition of ASM (ASM^+/−^) improves autophagy defects [6]. The main regulatory mechanism is the inhibition of lysosomal ASM and regulation of TFEB expression. In this study, PUE effectively suppressed ASM expression and decreased the degree of co‐localization between ASM and lysosomes (Figure S4A). This alteration may be attributed to the ability of PUE to hinder ASM trafficking to lysosomes, thereby modulating lysosomal function and autophagy. TFEB, a member of the MiT/TFE family of basic helix–loop–helix leucine zip transcription factors, serves as a pivotal regulator of lysosomal gene expression and function and orchestrates autophagy processes. Under normal conditions, TFEB is typically phosphorylated and sequestered in the cytoplasm. However, upon cellular starvation, TFEB is dephosphorylated and translocated to the nucleus, triggering the expression of downstream autophagic, lysosomal and other target genes. Consequently, modulating the phosphorylation status of TFEB and facilitating its nuclear translocation can significantly alter the expression of lysosomal, metabolic and other genes [33]. Notably, in our study, PUE effectively restored the nuclear entry activity of TFEB that was suppressed in the OGD cardiomyocyte model (Figure S4B), highlighting its potential therapeutic implications in regulating lysosomal function and autophagy. The enhancement of TFEB function can stimulate ALP function, promote protein clearance, and restore lysosomal biogenesis [34]. Subsequently, in the pathogenesis of neurodegenerative diseases [35], metabolic syndrome [36], cancer [37] and cardiovascular disease models [38, 39], ASM and autophagy dysfunction have been found to be closely associated. Recent studies have shown that using an ASM inhibitor or siRNA to inhibit ASM in high‐fat diet‐fed mice can reduce myocardial cell apoptosis and fibrosis and alleviate myocardial hypertrophy and heart dysfunction [40]. Therefore, we speculate that PUE may target ASM to regulate ALP function and protect the heart from HF‐caused pathological remodelling. Based on this hypothesis, we used siRNAs to inhibit ASM expression and examined autophagy. We found that autophagy flux and lysosomal expression levels were partially restored, and myocardial cell hypertrophy was suppressed under simulated myocardial ischemia conditions (Figure 6). This effect was consistent with the effect of a single PUE intervention and was more significant after additional PUE interventions, which verified our hypothesis. However, we did not verify whether PUE‐targeted ASM in vivo using ASM‐knockout animals. Further verification of the mechanism by which PUE targets ASM to regulate ALP function will be the focus of future studies. In summary, our data emphasised the potential of ASM‐targeting PUE to regulate the autophagy–lysosome axis to protect against HF.

**FIGURE 7:**
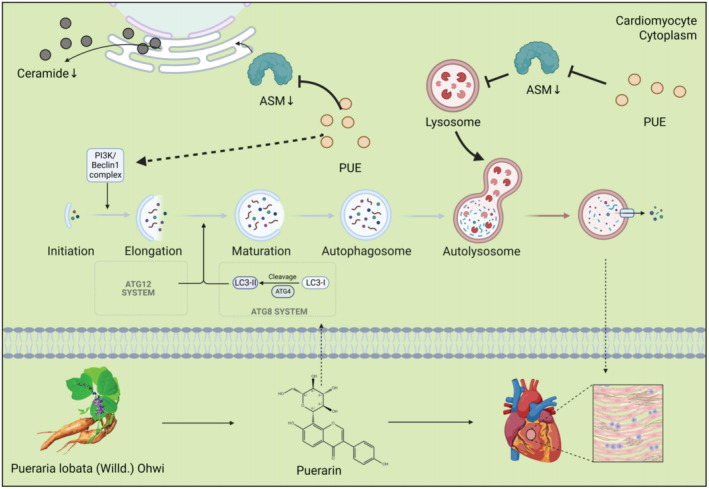
Mechanism of reversal of cardiomyocyte autophagy‐lysosomal dysfunction by Puerarin through inhibition of acid sphingomyelinase.

The mechanism by which ASM regulates autophagy is complex and varies according to the disease mechanism. Overall, our study demonstrated that PUE effectively improves cardiac function and protects cardiomyocytes from injury in mice with HF. The underlying mechanism appears to involve regulation of sphingolipid metabolism by PUE, specifically the downregulation of ASM expression and the inhibition of lysosome–ASM binding. Furthermore, PUE enhanced ASM‐mediated TFEB nuclear translocation, which subsequently modulated autophagy and the expression of lysosome‐associated proteins. These effects collectively preserve lysosomal function and ultimately restore autophagic flux, thereby contributing to the beneficial cardiac outcomes achieved (Figure 7), complementing the mechanism of PUE in treating HF and providing a possible target for treating HF.

## Author Contributions

**Yin‐ping Li:** investigation (equal), project administration (equal), validation (equal), writing – original draft (lead). **Qian He:** investigation (equal), methodology (equal), validation (equal). **Xiao‐ying Yu:** data curation (equal), software (equal), validation (equal). **Sheng‐tao Xiong:** data curation (equal), investigation (equal), software (equal). **Hong‐jing You:** data curation (equal), project administration (equal), software (equal). **Yue Xuan:** data curation (equal), software (equal), validation (equal). **Wei‐yan Liao:** investigation (equal), project administration (equal), software (equal). **Ze‐yu Chen:** data curation (equal), investigation (equal), software (equal). **Hai‐yan Li:** data curation (equal), investigation (equal), software (equal). **Wei Wang:** funding acquisition (equal), resources (equal), supervision (equal). **Yang Chen:** resources (equal), supervision (equal), writing – review and editing (equal). **Xiao Wang:** resources (equal), supervision (equal), writing – review and editing (equal).

## Conflicts of Interest

The authors declare no conflicts of interest.

## Supporting information


Figure S1

Figure S2

Figure S3

Figure S4

Figure S5

Appendix S1

## Data Availability

The data that support the findings of this study are available from the corresponding author upon reasonable request.
