# Supplementary figures and images for: Puerarin Reversing Autophagy‐Lysosomal Dysfunction via Acid Sphingomyelinase Inhibition in Cardiomyocytes
Source: J Cell Mol Med. 2025 Feb 24;29(4):e70427. doi: 10.1111/jcmm.70427 (PMC11850092; doi:10.1111/jcmm.70427)

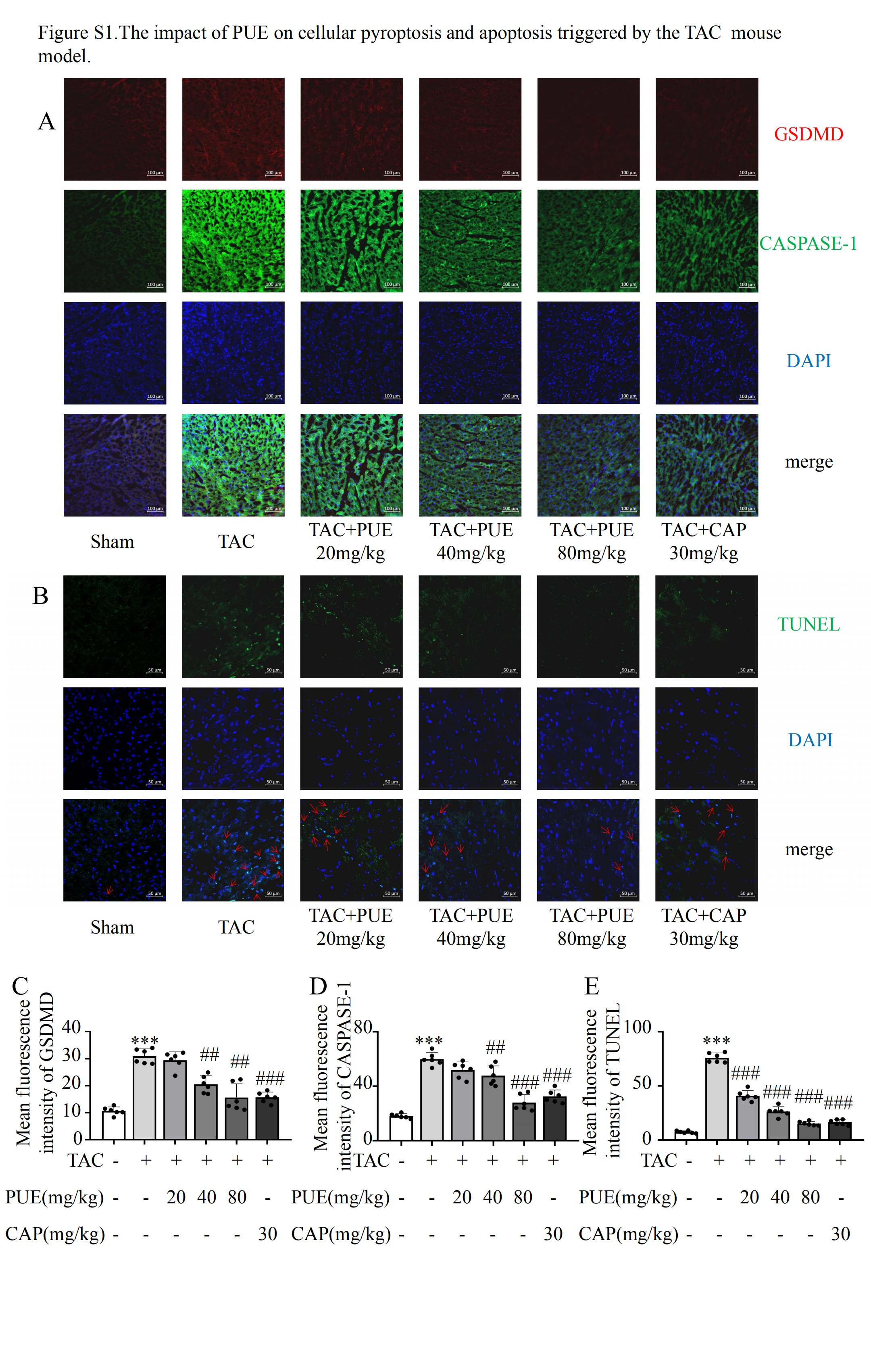

Supplement: Supplementary file 1 — Figure S1 [file JCMM-29-e70427-s006.tif]

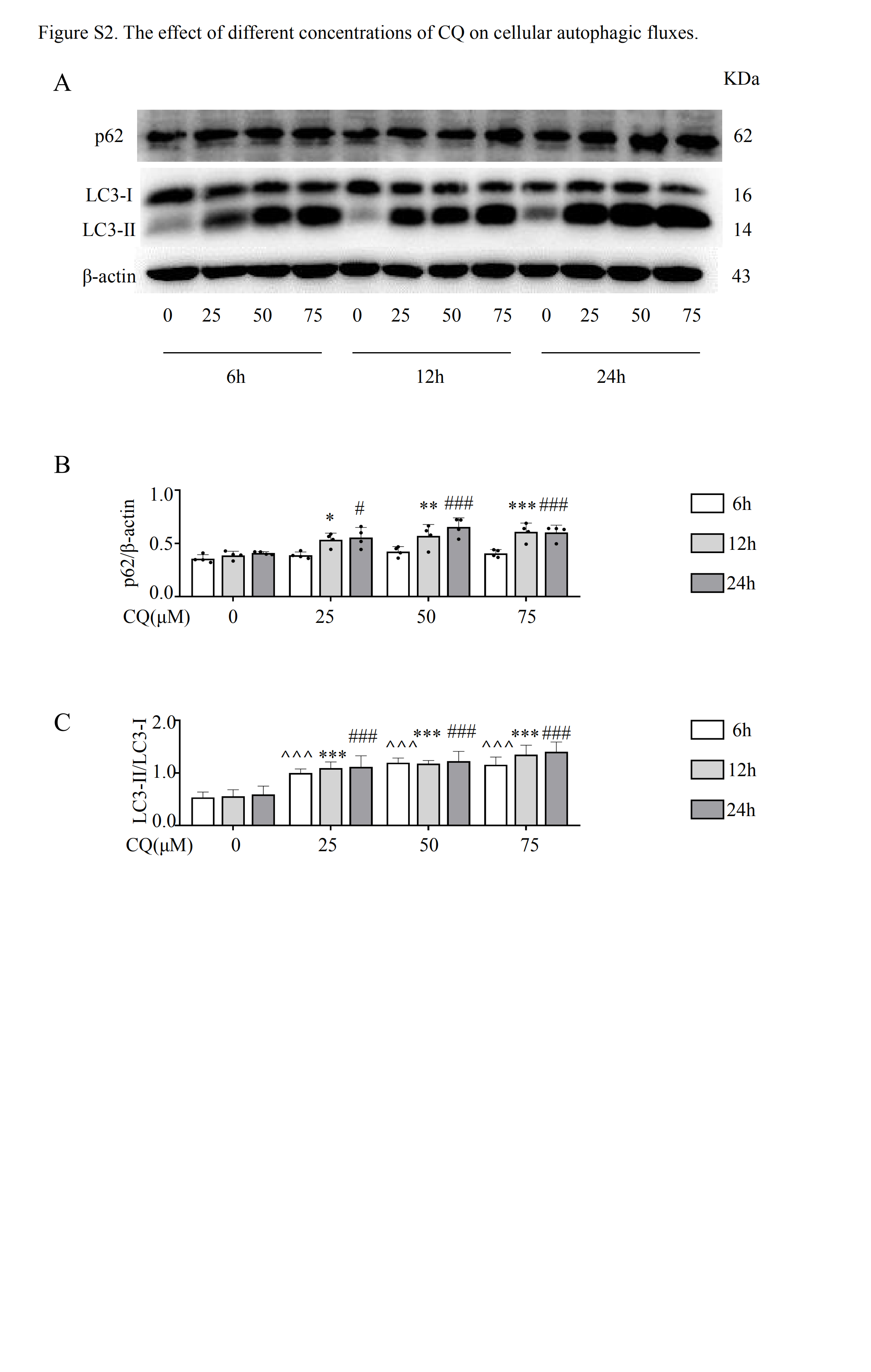

Supplement: Supplementary file 2 — Figure S2 [file JCMM-29-e70427-s002.tif]

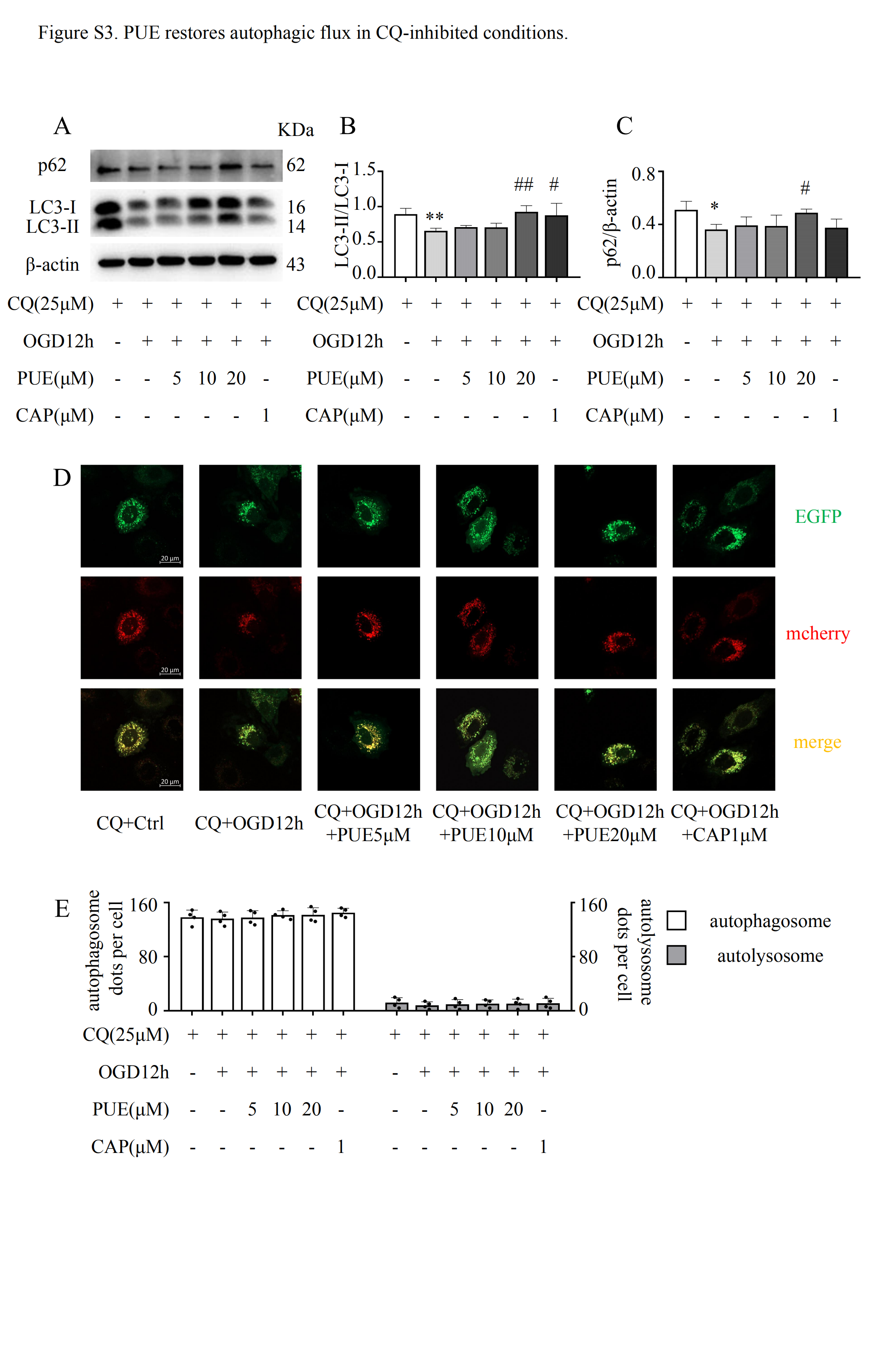

Supplement: Supplementary file 3 — Figure S3 [file JCMM-29-e70427-s004.tif]

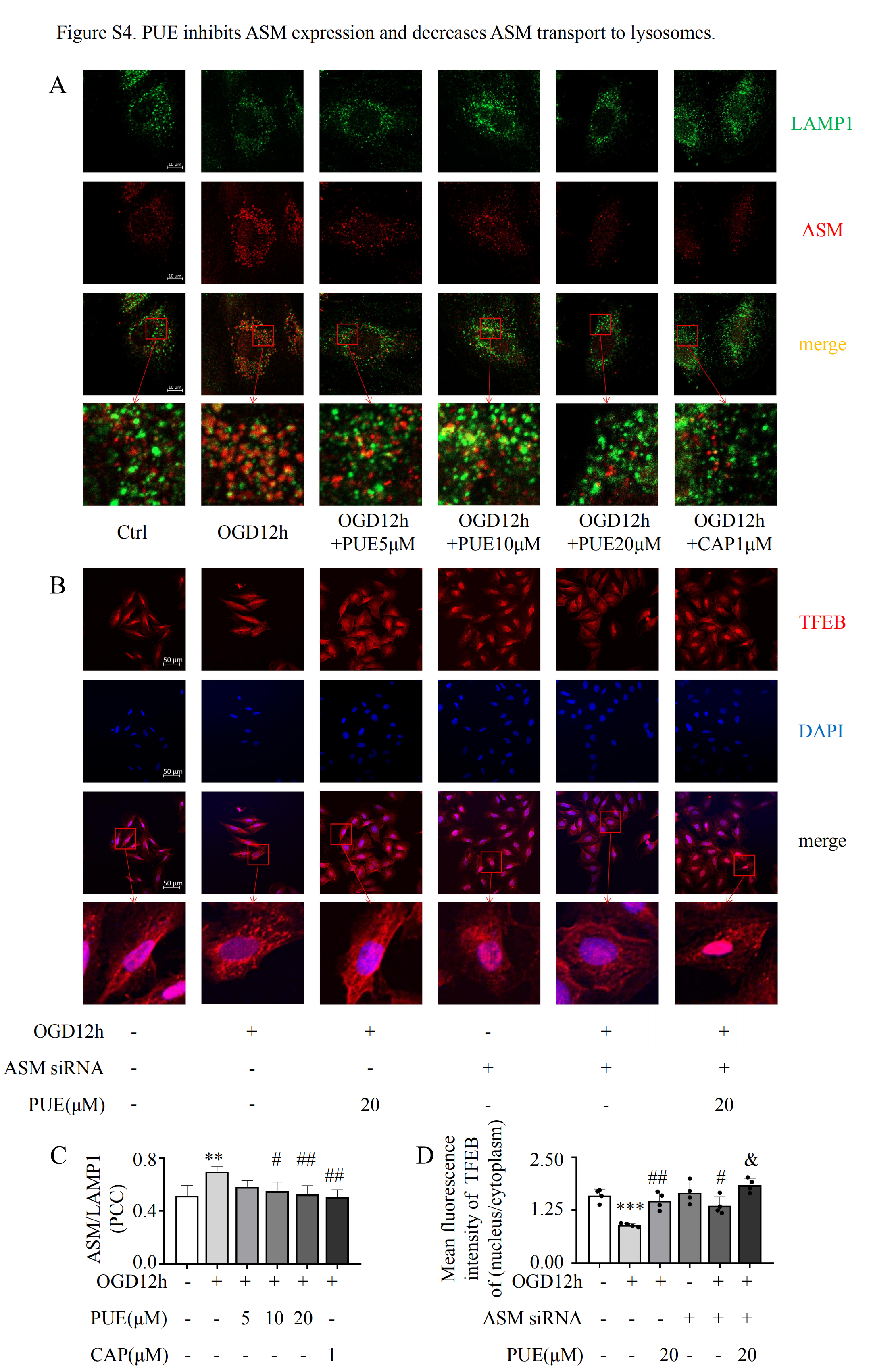

Supplement: Supplementary file 4 — Figure S4 [file JCMM-29-e70427-s001.tif]

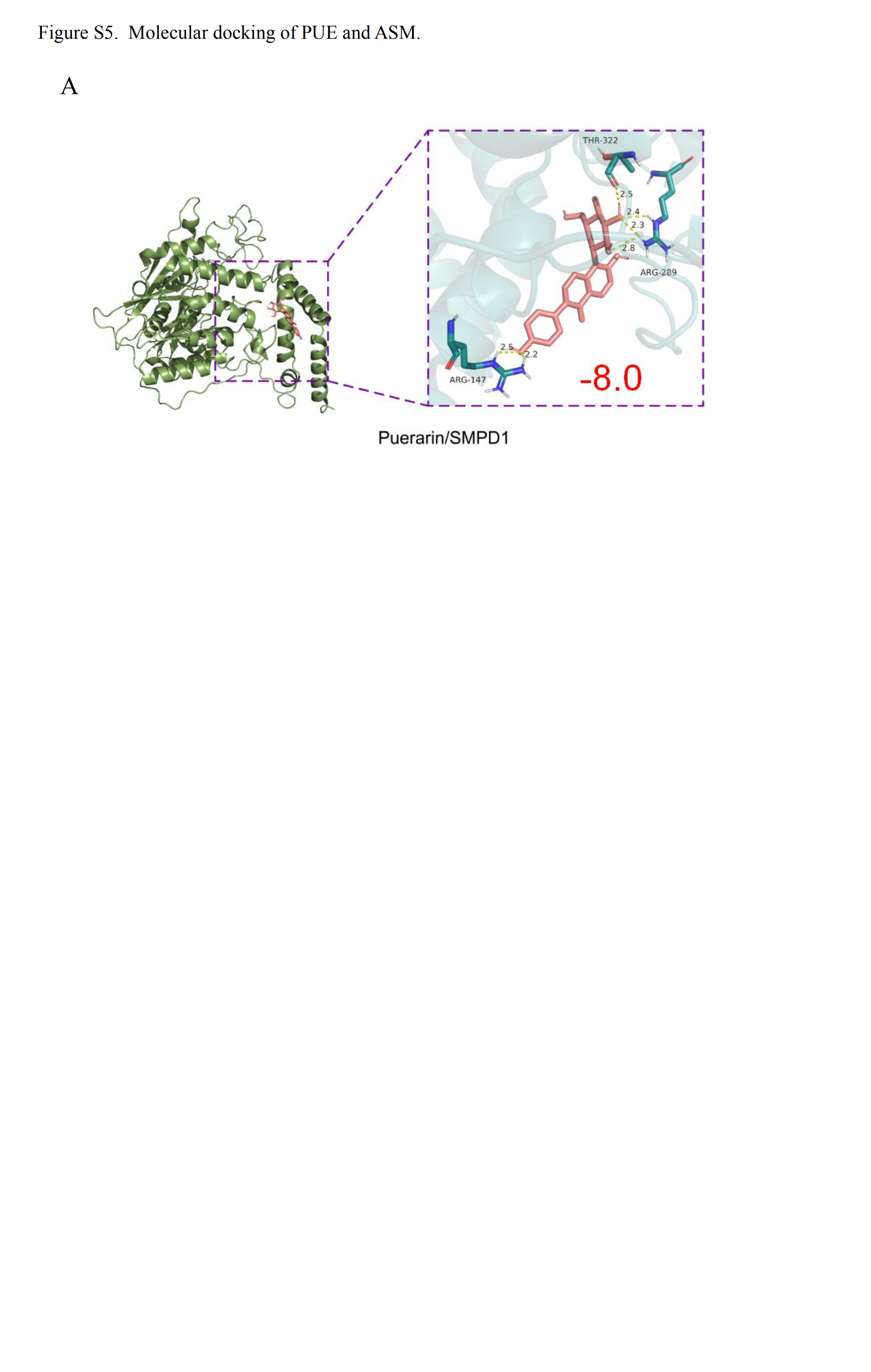

Supplement: Supplementary file 5 — Figure S5 [file JCMM-29-e70427-s003.tif]
