# Supplementary material for: Puerarin Reversing Autophagy‐Lysosomal Dysfunction via Acid Sphingomyelinase Inhibition in Cardiomyocytes
Source: J Cell Mol Med. 2025 Feb 24;29(4):e70427. doi: 10.1111/jcmm.70427 (PMC11850092; doi:10.1111/jcmm.70427)
Supplement: Supplementary file 6 — Appendix S1 [file JCMM-29-e70427-s005.docx]

**Appendix**

1. **Primer Sequences**

The following are the primer sequences used in this study:

**1.1 Mus (Mouse) Primer Sequences:**

BNP primer pair: Forward - GGAGAACACGGCATCATTGC; Reverse - CTCCAGCAGCTTCTGCATCT.

GAPDH primer pair: Forward - GAGCCTCCTCCAATTCAACCC; Reverse - GGGACGAGGAAACACTCTCC.

**1.2 Rat (Rat) Primer Sequences:**

ANP primer pair: Forward - CTGGGACCCCTCCGATAGAT; Reverse - CAATCCTACCCCCGAAGCAG.

BNP primer pair: Forward - CTGGGAAGTCCTAGCCAGTC; Reverse - AGGGCCTTGGTCCTTTGAGA.

GAPDH primer pair: Forward - GCATCTTCTTGTGCAGTGCC; Reverse - GATGGTGATGGGTTTCCCGT.

1. **SMPD1(ASM) siRNA Sequences**

The following are the siRNA sequences used in this study:

siSMPD1-1(siRNA-1)

Sense Strand：5’-GUCUCGACAAGAUCAGCUA-3’

Antisense Strand：5’-UAGCUGAUCUUGUCGAGAC-3’

siSMPD1-2(siRNA-2)

Sense Strand：5’-GUGCUACCACCUUUAUCAA-3’

Antisense Strand：5’-UUGAUAAAGGUGGUAGCAC-3’

siSMPD1-3(siRNA-3)

Sense Strand：5’-CACAGAUCCUGCUGGACAA-3’

Antisense Strand：5’-UUGUCCAGCAGGAUCUGUG-3’


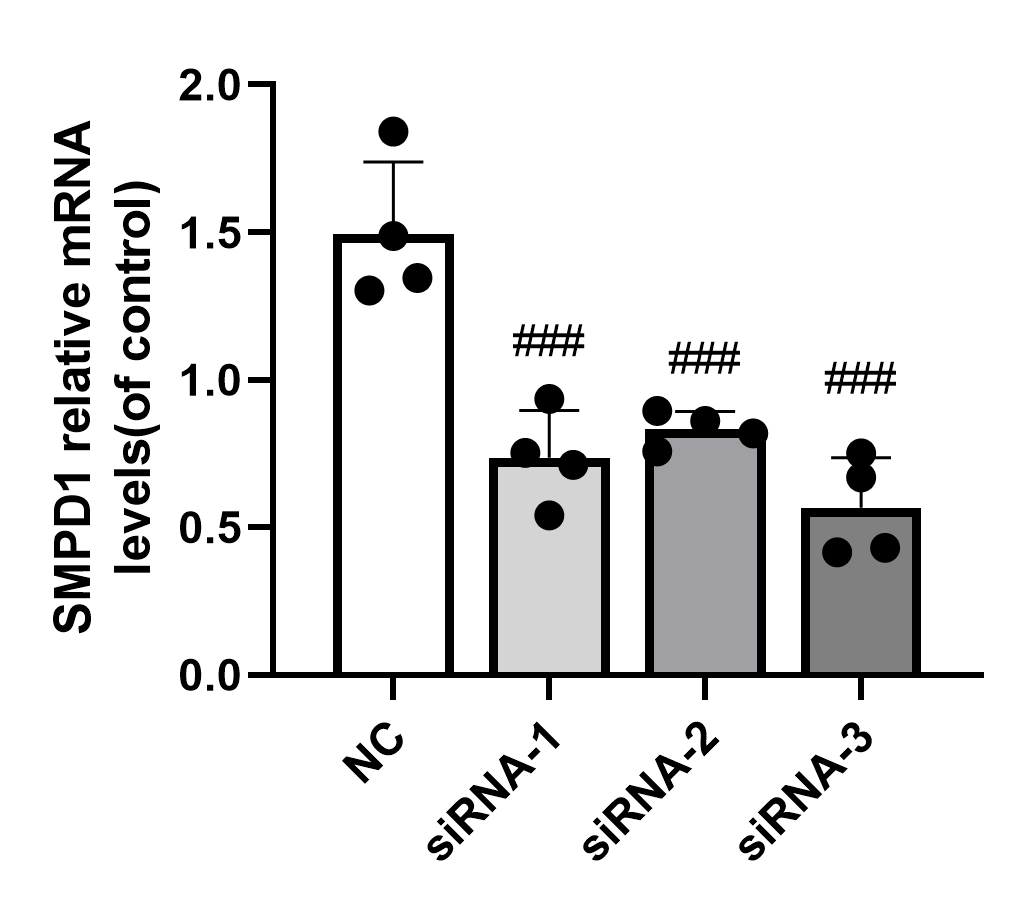


Figure 1: Detection of SMPD1 transcript levels after transfection of H9c2 cells using three different sequences of SMPD1 siRNA and determination of subsequent transfection of cells with SMPD1-3 (siRNA-3).
